# A Perspective on the Enhancer Dependent Bacterial RNA Polymerase

**DOI:** 10.3390/biom5021012

**Published:** 2015-05-21

**Authors:** Nan Zhang, Martin Buck

**Affiliations:** Department of Life Sciences, Imperial College London, Sir Alexander Fleming Building, Exhibition Road, London SW7 2AZ, UK; E-Mail: nan.zhang@imperial.ac.uk

**Keywords:** RNA polymerase, Sigma54, transcription, AAA+ ATPase

## Abstract

Here we review recent findings and offer a perspective on how the major variant RNA polymerase of bacteria, which contains the sigma54 factor, functions for regulated gene expression. We consider what gaps exist in our understanding of its genetic, biochemical and biophysical functioning and how they might be addressed.

## 1. Introduction

Along with the recognition that bacterial RNA polymerases were heterogeneous with respect to their sigma factor content, came the finding that two classes of sigma factor existed in many different types of bacteria [1]. Unlike the major sigma70 class, the sigma54 class of factor was distinctive in being enhancer dependent and relying on a specialised class of transcription activator which used ATP binding and hydrolysis to catalyse the formation of open promoter complexes (RP_O_). Closed promoter complexes (RP_C_) rarely spontaneously isomerised to open complexes, and so sigma54 dependent systems showed a typical major dependence on cognate activators (reviewed in [2,3]). No hard and fast rules allow the prediction of which bacteria will contain an *rpoN* gene or genes (some organisms have two *rpoN*s) encoding sigma54, although informatics suggests sigma54 use is rooted in the control of cell envelope functions in response to stress [4]. Because the sigma54 factor controls important bacterial stress response genes in pathogenicity and in agriculture [5,6,7,8,9], there is considerable interest in working out where the dependence arises in biochemical and structural biology terms. Further, what are the advantages the system may have over conventional repression and activation systems used by the sigma70 class of RNA polymerase holoenzymes?

## 2. The Sigma54 Factor

Work from Sydney Kustu’s lab provided the first biochemical evidence that the sigma54 factor (encoded by *rpoN*, also called ntrA) was a dissociable sigma factor which directed RNA polymerase as a holoenzyme to the −12, −24 promoter sequences from which it transcribed [10,11]. Sequence analysis of a range of *rpoN* encoded genes was to provide clear indications that the sigma54 protein was unrelated to the major sigma70 class of sigma factors at the level of primary sequence and most likely its fold and tertiary structure. For the most part this lack of relatedness has been upheld by what limited structural data sets for fragments of sigma54 have been obtained to date. The lack of relatedness suggests that sigma54 may direct a different set of structural transitions in taking RPc to RPo to that orchestrated by sigma70 type factors, rather than just changing the kinetics of the same pathway of conformational change.

**Figure 1:**
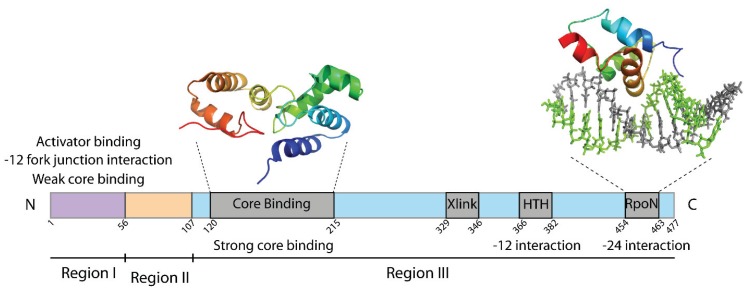
Domain organisation of *Escherichia coli* σ^54^. The DNA binding function is mainly associated with σ^54^ Region III, including the DNA crosslinking motif (Xlink), a putative helix-turn-helix motif (HTH) for −12 recognition via E378 and the RpoN box for −24 binding. Structures of two σ^54^ Region III fragments have been obtained (PDB entries: 2K9L and 2O8K).

Sigma54 contains two highly conserved domains (Regions I and III, Figure 1), separated by what may be a flexible linker (Region II). The glutamine- and leucine-rich Region I interacts with activator ATPases, core RNAP and the −12 promoter sequence, thus posing an energy barrier to spontaneous isomerization of RPc to RPo. Region I mutations and deletion often resulted in activator-bypass phenotypes [12,13]. Sigma54 Region II is dispensable for RNAP isomerisation and interaction with DNA. Indeed many bacterial sigma54 proteins do not naturally contain this domain, arguing for its ancillary role. Sigma54 Region III is primarily involved in binding to the promoter DNA at several sites, with the strongest interaction being between the RpoN box and the −24 promoter element. The RpoN box forms a winged helix-turn-helix (HTH) motif and inserts the recognition helix into the major groove of the −24 promoter sequence [14,15]. Mutations in the RpoN box have been shown to cause a more than 80% reduction in promoter DNA binding affinity [16].

## 3. Promoter Recognition

Apparently sigma54 RNAP finds promoters by a direct binding route and not sliding, at least *in vitro* [17]. At its cognate promoters the holoenzyme opens up the A:T base pair 3' to the promoter −12 consensus GC sequence and so a repressive fork junction structure with which the Region I of sigma54 interacts is created. Resolution of this structure by the activator ATPases is a part of the enhancer dependent activation of the RP_C_ to yield an RP_O_. Details of the −12 recognition problem and how the local −12 proximal un-stacking is achieved in the stable RP_O_ from an unstable prior fully stacked DNA RP_C_ [18] is now a structural biology issue. Genetics implicates a HTH motif E378 residue and specifically also residue L25 in sigma54 in the −12 GC recognition, but the specific amino acid sequences in sigma54 and in core enzyme needed for base un-stacking just downstream of the −12 GC are unknown although may well map to the Region I of sigma54. Similarly DNA melting defective mutants (for RP_O_) of sigma54 have not been obtained, in contrast to sigma70, and so quite how sigma54 contributes to opening DNA from −10 to −1 is unknown. However mutants in sigma54 able to allow RP_O_ formation *in vitro* without activation suggest the barrier to RP_O_ formation is distinct from determinants that allow formation and maintenance of (at least unstable) forms of RP_O_. Although not widely surveyed across promoters, it seems that the sigma54 holoenzyme forms fewer abortive RNA products before elongation than does the sigma70 containing enzyme [19], and so features of sigma70 contributing to the frequency of abortive initiation may not have counterparts in sigma54.

In contrast to a complex recognition of the promoter −12 intimately linked to maintaining RP_C_, recognition of the −24 promoter sequence seems relatively well understood, and involves a HTH motif and the RpoN box amino acid sequence, as solved by the Wemmer group by NMR [14]. In contrast to the −35 recognition of sigma70 promoters being mediated by the flap domain of the core RNAP, no such dependence seems so for sigma54 although other roles for the flap domain are suggested by promoter DNA footprinting experiments with a flapless core and sigma54 [20].

## 4. Activator Remodel of the Sigma54 Holoenzyme

**Figure 2:**
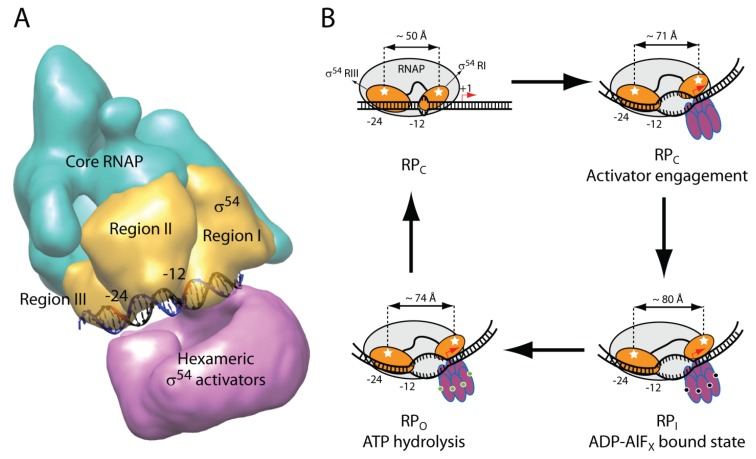
Domain architecture and the proposed mechanism of activation. (**A**) Cryo-EM reconstitution of the Eσ^54^-PspF-ADP-AlF_x_ with the promoter DNA modelled in [21]; (**B**) Domain movements of σ^54^ Regions I and III during the transcription activation cycle (modified from [24]). The white stars depict fluorophores used in the smFRET experiments.

Sigma54 activators (such as NtrC and PspF) belonging to the AAA+ protein family assemble into hexamers and fuel the rearrangement of RP_C_ to RP_O_. Cryo-EM reconstitutions carried out by the Zhang lab revealed up to three sigma54 activators within a hexamer could directly contact the RP_C_ structure for isomerisation [21]. These contacts were made asymmetrically via the GAFTGA loop one motifs to sigma54 Region I and the upstream −30 promoter region [22], and were possibly accompanied by the splitting of the hexameric ATPase ring in order to exert directional forces [23]. A bridging density within the holoenzyme was observed to physically block the DNA loading channel formed between the β and β’ subunits prior to activation (Figure 2A). This density was assigned to a part of sigma54 Region I and it relocated downstream towards the +1 site in the intermediate complex (RP_I_) when the ADP-AlF_x_ hydrolysis analogue was added. The DNA melting site was misaligned with the DNA loading site in the RP_I_ in the proposed model, which could constitute an ancillary inhibitory mechanism. The Stockley and Tuma labs further addressed sigma54 domain movements in relation to promoter DNA and ATP hydrolysis by smFRET analysis (Figure 2B, [24]). Sigma54 Region I moved by approximately 9 Å towards the leading edge of the −10 to −1 transcription bubble in the RP_I_ when activated with ADP-AlF_x_. This downstream movement upon activation fully agreed with the Cryo-EM observations and potentially correlated with blockage removal by the “power stroke” action of activator ATPases. Once ATP was fully hydrolysed, Region I would retract slightly upstream, possibly to accompany the DNA loading event. In contrast, Region III remained rather static with respect to the −24 promoter sequence along the activation pathway for making RP_O_ from RP_C_.

Overall it is clear that activation for making RP_O_ requires a repositioning of the sigma54 Region I, which occurs through the use of at least two GAFTGA loops of the activator ATPases that bind there. Whether the activator binds a rare state of the holoenzyme in RP_C_, or drives the formation of an entirely new functional state is not yet clear. However, the R336A sigma54 variant phenocopies the action of the binding of the activator to RP_C_ in that the Region I has moved compared to that in RP_C_, and so the barrier to passing from RP_C_ to at least an RP_I_ if not RP_O_ is not so large. Release of the activators from the sigma54 Region I may be key in passing to RP_O_ from RP_I_, as may a contact of the activator with upstream promoter DNA. Kinetic studies on the *glnAP2* promoter using COSMO methodologies indicates that the transition from RP_C_ to transcript generation takes around ninety seconds, and activators interact with both RP_C_ and RP_O_ [18]. These single molecule studies also lend support to the idea that an initial form of RP_C_ exists without the DNA base unstacking at −12 [18]. Given the heterogeneous nature of nucleotide bound states of the activators, that the hexameric activators may assume an opened form and use a subset of the six possible sigma-contacting mobile surface features [22,25]. Their asymmetric functioning seems a necessary part of the transcription activation mechanism and may reflect the asymmetric target of the σ^54^ within the RP_C_. A processive functioning of the activators may not be necessary for activation, because the activator bound RP_I_ has been shown to possess RP_O_ like characteristics [26]. However quite how and when DNA opening occurs for making the RP_O_ with sigma54 is not known, and could involve other functions of the activators ATPase rather than a direct sigma DNA melting activity. Notably when double stranded DNA is outside of the RNAP in RP_I_, extensive cross links can be made with the bound activator, which might guide dsDNA into the holoenzyme for making RP_O_, or indeed act on it directly for making RP_O_ [21]. An interaction of DNA with the activators ATPase domain has been observed [27]. Further, activator binding to RP_C_ (enabled by the use of non-hydrolysable nucleotide analogues such as ADP-AlF_x_) when promoter DNA from −10 to −1 is unpaired allows transcription initiation, suggesting the holoenzyme can accept ssDNA and then open up the start site to allow RNA synthesis, without the need for ATP hydrolysis by the activator protein [26].

## 5. Signaling for Sigma54 Dependent Transcription

Genes under control of the sigma54 are required under specific stress conditions or when a particular C or N source is available. Classical examples include the NtrC dependent activation of genes in response to nitrogen limitation, the use of XylR in expressing genes for catabolism of aromatic hydrocarbons and PspF in activating the psp response for inner membrane stress. Although all contain the critical AAA+ domain needed for remodelling the closed promoter complex, the activators are all signaled to via a range of N-terminal regulatory domains. These fall into a wide range of different classes ranging from the response regulator type aspartic acid phosphorylation target in two component members, to GAF domains in NifA and CARF domains in RtcRs (reviewed in [2]). In many cases the exact ligand interacting with these N-terminal regulatory domains remains unknown.

## 6. Conclusions and a Final Perspective

Detailed structural insights into RP_C_, RP_I_ and RP_O_ are now needed to work out quite what it is in structural terms that establishes the barrier to making RP_O_, and how RP_C_ and RP_O_ interconvert. Comparisons to the use of the sigma70 Region I as a place keeper for DNA and that of sigma54 Region I as a target for activation, maintaining the repressive fork junction structure and escaping the action of certain phage inhibitors of RNAP activity such as T7 phage gp2 will be one valuable outcome from such studies. Similarly, knowing what the functional state the core RNAP is in when bound to sigma54 will provide insights into the importance of the sigma-core interface in gene control and potentially antibiotic action. For example in the presence of sigma54 is the core enzyme in a catalytically competent state, and can smFRET data be reconciled with RNAP clamp opening and clamp closing, and the processive closed state of the core enzyme? Do the sigma54 and its activators take the RNAP down a new set of conformationally distinct changes for making RP_O_, or is it simply altering the kinetics of a single pathway for making the RP_O_ from the RP_C_?

Quite where sigma54 came from remains a mystery-it cannot be rooted and seems to have no obvious structural counterpart (although detailed structural analysis may overturn this view, if not for the full protein then for some of its domains). Genomics methods and RNAseq studies will now no doubt offer us perspectives on why have sigma54 at all-is it a relic of a transcription repression mechanism, can it (or has it?) evolve in some cases to activator independence, and does it have repressive functionality alongside its gene activation responsiveness? Currently ChipSeq and RNAseq work with sigma54 and its holoenzyme to define its regulon suggests complexity in the roles of sigma54 [28,29]. For example some RP_C_s seem not to be served by cognate activators, and amongst these a subset seem to repress transcription by other forms of RNAP holoenzyme. In combination with genomic approaches to better understand roles of sigma54 binding sites on bacterial chromosomes, sufficient knowledge of sigma 54 and sigma70 structure-function relationships will allow us to make orthogonal non-native chimeric sigma factors-to tackle issues of promoter use in synthetic biology and an increased insularity from the bacterial chassis used in such work. Since sigma54 is served by cognate activators of the AAA+ protein family, detailed atomic structures of their co-complexes with RP_C_ will help work out how such AAA+ proteins remodel their targets. Lastly, the full role of sigma54 in bacterial physiology is unknown. Many of its activators respond to signals which are poorly characterised, yet underpin important processes such as RNA repair (the *rtcBA* genes activated by RtcR) and envelope maintenance (the *psp* operon activated by PspF). As details of how genes turn on and off in individual cells through transient changes in, for example, local DNA superhelicity to yield the so called bursty behaviours of gene transcription revealed by time series studies, one might wonder if the sigma54 factor with its coupled ATPase overlays some special features to such time series.
